# Supplementary material for: Genome graphs reveal the importance of structural variation in Mycobacterium tuberculosis evolution and drug resistance
Source: Nat Commun. 2025 Nov 28;16:10746. doi: 10.1038/s41467-025-65779-9 (PMC12663572; doi:10.1038/s41467-025-65779-9)
Supplement: Supplementary file 2 — Description of Additional Supplementary File [file 41467_2025_65779_MOESM2_ESM.pdf]

**Description of Additional Supplementary Files**

**Supplementary Data 1**: Long-read data accession IDs in NCBI of public and newly-sequenced isolates included in the <i>Mtb</i>-PRG

**Supplementary Data 2**: Quality metrics of all long-read assemblies included in the PRG.

**Supplementary Data 3:** Differential expression results of all <i>Mtb</i> genes comparing an L1.2.1 isolate against L1.1.1 and L1.1.2 isolates.

**Supplementary Data 4**: Significantly associated <i>Mtb</i> SVs with drug resistance.

**Supplementary Data 5:** Significantly associated <i>Mtb</i> SVs that fall in <i>PE/PPE</i> genes with drug resistance.

**Supplementary Data 6**: Significantly associated <i>Mtb</i> genes with drug resistance.
